# Fluorescent TAPY–bodipy dyads as tools for imaging fungal mitochondria by confocal microscopy and flow cytometry

**DOI:** 10.1007/s00216-026-06404-6

**Published:** 2026-02-26

**Authors:** Jean C. Neto, Rosa de Llanos, Francisco Galindo

**Affiliations:** 1 Departamento de Química Inorgánica y Orgánica, Universitat Jaume I de Castellón, Avda. Vicente Sos Baynat S/N, 12071 Castellón de La Plana, Spain; 2 Unidad Predepartamental de Medicina, Universitat Jaume I de Castellón, Avda. Vicente Sos Baynat S/N, 12071 Castellón de La Plana, Spain

**Keywords:** Mitochondria, Imaging, Fluorescence, Microscopy, Flow cytometry, Targeting, *Candida albicans*, *Candida krusei*, Fungi, Yeast, Bioanalysis

## Abstract

Mitochondria-targeted fluorescent probes are essential tools in cell biology, yet most have been optimized for mammalian cells rather than fungi, despite the growing clinical relevance of pathogenic yeasts. Here, we evaluate six triarylpyridinium–bodipy (TAPY–BDP) conjugates as mitochondrial markers in *Candida albicans* and *Pichia kudriavzevii* (formerly *Candida krusei*), using a triphenylphosphonium–bodipy (TPP–BDP) dyad and a non-targeted propyl–bodipy (prop-BDP) as references. Confocal microscopy and flow cytometry quantified uptake and localization. Colocalization with MitoTracker™ Deep Red gave Pearson correlation coefficients up to 0.9, supporting efficient targeting for most TAPY derivatives. TAPY–BDPs with R = H, Me, OMe, or NMe_2_ showed higher mitochondrial fluorescence than the more lipophilic analogues (R = Cl, CF_3_) and the TPP–BDP reference at 500 nM, consistent with flow cytometric analysis. Notably, TAPY(OMe)–BDP maintained robust mitochondrial staining at 100 nM in both fungi, whereas TPP–BDP decreased to near-background. Carbonyl cyanide 3-chlorophenylhydrazone (CCCP) reduced fluorescence for all cationic probes, confirming mitochondrial membrane potential–driven accumulation. Overall, these results indicate that TAPY is a competitive alternative to TPP for mitochondrial vectorization in fungi, expanding probe options for bioanalytical studies in azole-resistant yeasts and potentially enabling mitochondria-directed therapeutic delivery.

**Graphical abstract:**

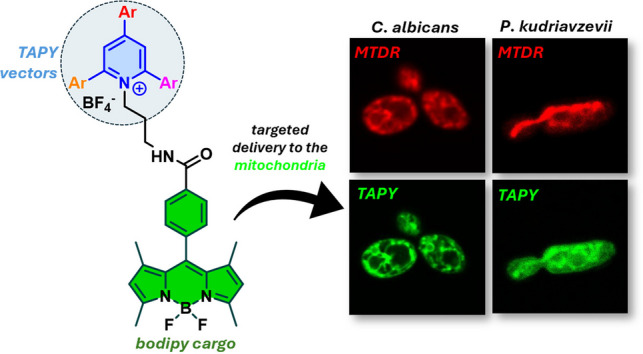

**Supplementary Information:**

The online version contains supplementary material available at 10.1007/s00216-026-06404-6.

## Introduction

Mitochondria are complex cellular organelles that play pivotal roles in eukaryotic cells, extending far beyond energy generation via oxidative phosphorylation [1]. Accumulating knowledge about these organelles has led to a more comprehensive understanding of numerous pathologies and corresponding therapeutic interventions [2–5].

To gain this knowledge, fluorescence-based analytical techniques such as microscopy [6] and flow cytometry [7] have been indispensable. However, these techniques rely on chemical tools (fluorochromes) that selectively label mitochondria. As a result, mitochondrial fluorescent probes have evolved in recent years from simple dyes to complex molecular architectures specifically designed for this bioanalytical purpose. Numerous examples of carefully engineered mitochondrial probes can be found in the specialized bioanalytical literature. Most research developing mitochondrial probes has focused on mammalian cells, particularly in relation to cancer and other diseases [8–23]. In contrast, much less attention has been paid to fluorescent imaging mitochondria in other cells that also contain these organelles, such as fungi [24, 25]. Given that fungal pathogens represent an emerging threat to public health systems, more effort should be directed toward understanding the biology of these potential hazards [26].

Mitochondrial dyes developed for mammalian cells have been applied in fungal studies. For instance, nonyl acridine orange (NAO) [27, 28] rhodamine 123 (Rh123) [29], MitoTracker Green (MTG) [30], and DiOC_6_ [31], as well as designed conjugates to investigate drug mechanisms of action [32–35]. However, the number of studies on mitochondrial imaging in mammalian cells remains disproportionately higher than those focused on fungi [36–41].

One reason for this imbalance may lie in the substantial societal interest in cancer and other human pathologies, despite the growing but underrecognized threat of microbial infections. Another contributing factor could be the intrinsic difficulty of delivering fluorescent probes into fungal cells, which possess a thick cell wall composed of chitin, β-glucans, and mannoproteins, structures absent in mammalian cells [42]. Additionally, fungal cells frequently express efflux pumps that expel toxic molecules [43], including fluorescent probes. While efflux pumps also exist in mammalian cells [44], fungi exhibit a particular proficiency in developing such resistance mechanisms [45]. Indeed, several established assays for assessing efflux pump activity in yeasts are based on mitochondrial dyes such as NAO [46], Rh123 [47] and rhodamine 6G (Rh6G) [48–51], which are expelled from fungal cells rather than retained.

**Fig. 1:**
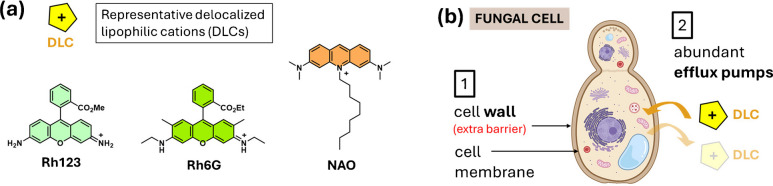
**(a)** Examples of mitochondrial probes based on lipophilic cations; **(b)** schematic illustration of the barriers, present in fungal cells, for the uptake of probes. Cell illustration created with BioRender.com

Most mitochondrial fluorescent probes reported to date are lipophilic cations, as the strong negative membrane potential of mitochondria (− 150 to − 180 mV) drives their accumulation into the organelle. This accumulation is further enhanced by the high affinity of these probes for the lipid-rich inner mitochondrial membrane [52, 53]. This principle underlies the utility of classical dyes such as NAO, Rh123, MTG, Rh6G, DiOC_6_, and analogs. Moreover, this concept has been leveraged to design mitochondrial targeting carriers for delivering both fluorescent probes and therapeutic molecules. A prominent example is the triphenylphosphonium cation (TPP), the paradigmatic mitochondrial vector [54]. In Fig. 1, it is presented in a graphical manner the basic concepts above described.

We have recently described a new family of mitochondrial carriers that resemble the structure of triphenylphosphonium (TPP) but offer significantly broader synthetic versatility. These carriers are based on a triarylpyridinium (TAPY) core. Notably, these structures have been successfully employed to deliver fluorescent cargoes, such as bodipyfluorophores, to the mitochondria of cancer cells [55].

Given the increasing interest in imaging the mitochondria of pathogenic fungi, we sought to investigate whether this new class of cationic mitochondrial probes could also be applied to stain fungal mitochondria. If so, a further question would be to determine the structural features within the TAPY framework that are essential for optimal performance in fungal cells. Here, we present the results of this investigation.

## Materials and methods

### Probes stocks solutions

Probes **TAPY(H)-BDP, TAPY(Me)-BDP**, **TAPY(OMe)-BDP**, **TAPY(NMe**_**2**_**)-BDP**, **TAPY(Cl)-BDP,** **TAPY(CF**_**3**_**)-BDP**, **TPP-BDP**, and **prop-BDP** were synthesized as described previously [55].

### Yeast strains and staining procedures

Yeast strains (*Candida albicans*, CECT 1394 and *Pichia kudriavzevi*,CECT 19105) used in flow cytometry and confocal fluorescence microscopy assays were initially cultured by streaking from glycerol stocks onto Sabouraud Dextrose Agar (SDA) (Scharlab) plates and incubated at 37 °C for 24 h. Subsequently, an inoculum was prepared in 1 × Sabouraud Broth (Scharlab) and incubated at 37 °C for 24 h under agitation. Cells were harvested by centrifugation and washed three times with phosphate-buffered saline (PBS, pH 7.4) to remove residual culture medium. The final cell suspension was adjusted to a concentration of 1 × 10^6^ cells/mL in PBS. Aliquots of this suspension were distributed into microtubes for individual treatments [56]. Each treatment involved the addition of **TAPY-BDP**, **prop-BDP**, or **TPP-BDP** probes at a final concentration of 500 nM or 100 nM, along with Mitotracker^TM^ Deep Red FM at 100 nM. The cells were incubated at 37 °C for 30 min, followed by centrifugation at 10,000 rpm for 5 min and three successive PBS washes to eliminate non-internalized probes. After the final wash, cells were resuspended in PBS and immediately prepared for flow cytometry and confocal microscopy analysis.

### Confocal laser scanning microscopy (CLSM)

For CLSM image acquisition, after the staining and washing steps described previously, samples were resuspended in 50 µL of PBS, and a drop of this suspension was added onto the pre-deposited mounting medium (90% glycerol in PBS) at the center of each slide [29, 57]. The cells were then covered with a coverslip and sealed with topcoat. Images were acquired using a Leica SP8 confocal microscope equipped with a 60 × oil immersion objective. The excitation and emission settings for each channel were as follows: bright field; for the mitochondrial probes (**TAPY-BDP**, **TPP-BDP**, and **prop-BDP**), excitation was performed with a 488 nm laser and emission was collected between 500 and 550 nm; for MitoTracker Deep Red FM, excitation was performed with a 633 nm laser and emission was collected between 650 and 750 nm. After image acquisition, the data were first processed using LAS X Office. Quantitative fluorescence analysis was performed using ImageJ (Fiji). For each image, regions of interest (ROIs) corresponding to individual cells were manually delineated using the polygon selection tool, ensuring random field selection and consistent treatment of all samples. The same background ROI, selected from a cell-free region of each image, was used for all measurements within that image. For each ROI, the area (A), mean gray value (MGV), and integrated density (ID) were obtained. The corrected total cell fluorescence (CTCF) was then calculated as CTCF = ID − (A × mean background). This correction minimizes the influence of background noise, variations in cell size, and providing a more reliable estimation of intracellular fluorescence intensity. This approach is widely recognized as a robust semi-quantitative method for microscopy-based fluorescence analysis [16, 55, 58]. Statistical analysis of fluorescence data was carried out using GraphPad Prism 10. Data distribution and variance homogeneity were assessed using the Anderson–Darling, D’Agostino–Pearson, Shapiro–Wilk, Kolmogorov–Smirnov, Brown–Forsythe, and Bartlett tests (Table S1). When assumptions were met, one-way ANOVA followed by Dunnett’s multiple comparisons test was applied, using **TPP–BDP** as the reference (Table S2). Residual diagnostics were evaluated using Q–Q and residual-versus-predicted plots (see ESI, Figure S1 and Table S1). In cases of deviation from normality or homoscedasticity, the Kruskal–Wallis test followed by Dunn’s multiple comparisons test with false discovery rate (FDR) correction was used (Tables S3–S4). Results are expressed as mean ± SD or median ± interquartile range (IQR), with significance levels indicated as ****p < 0.0001, ***p ≤ 0.001, **p ≤ 0.01, *p ≤ 0.05, and ns = not significant.

### Flow cytometry

After the growth and staining protocol of *C. albicans* cells, the microtubes were analyzed using a BD Accuri™ C6 flow cytometer, following the method described by Ludovico et al. with some modifications [29]. For the probes **TAPY-BDP** and **TPP-BDP**, fluorescence was detected in the FL1-H channel, using a 488 nm laser for excitation and a 533/30 nm emission filter (FITC/GFP settings). The green fluorescence signal from the probes was three orders of magnitude higher than the autofluorescence of the control cells (without any probe). For each experiment, approximately 12,000 events were recorded. Flow cytometry assays were performed in three independent biological replicates, each analyzed under identical instrument settings. The representative histograms shown in Fig. 5a correspond to a single experiment, while the quantitative bar chart in Fig. 5b represents the mean ± SD of the three biological replicates. The acquired data were processed and visualized using the open-source software Floreada.io (https://floreada.io/analysis).

### Mitochondrial membrane potential assay

To determine whether the uptake of the **TAPY–BDP** conjugates depends on mitochondrial membrane potential, a depolarization assay was carried out using the protonophore CCCP as described by López-Fernández et al*.*, with some modifications [59]. The procedure followed the same culture, staining, and incubation conditions described above for the confocal and flow cytometry experiments. Briefly, *C. albicans* cells were prepared at 1 × 10^6^ cells/mL and incubated with each **TAPY-BDP** probe at 100 nM in the presence or absence of 50 µM CCCP, which served as the mitochondrial depolarizing agent. Samples were incubated for 30 min at 37 °C under identical conditions to the standard staining protocol. After incubation, cells were washed three times with PBS and immediately analyzed by flow cytometry using the FL1-H channel (488 nm excitation; 533/30 nm filter). A reduction in fluorescence intensity in the + CCCP samples relative to the –CCCP condition was interpreted as evidence of membrane potential–dependent accumulation. DiOC_6_ was included as a positive control for potential-dependent mitochondrial staining.

## Results and discussion

### Studies with *Candida albicans* at 500 nM (probe concentration)

**Fig. 2:**
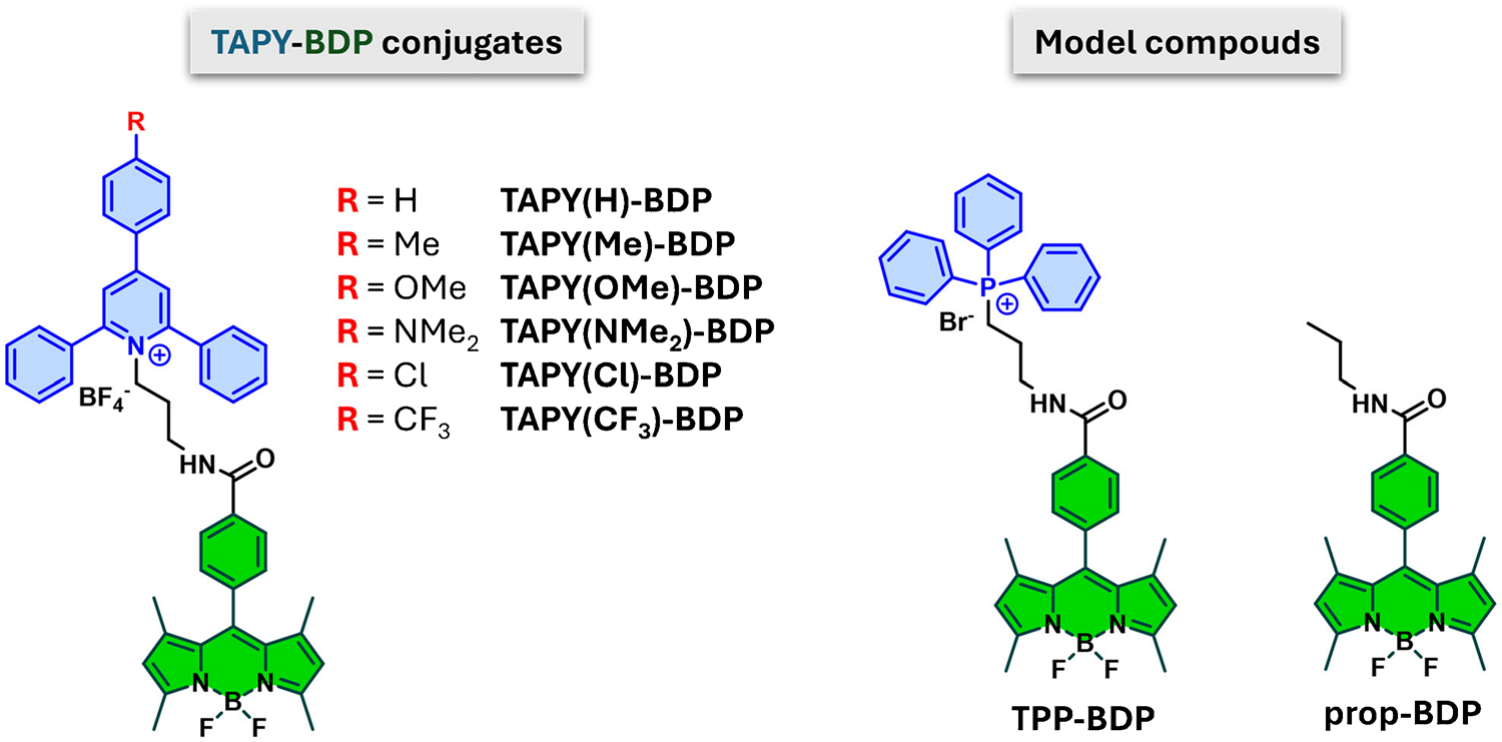
Chemical structures of the **TAPY-BDP** conjugates investigated in this study, and the model compounds **TPP-BDP** and **prop-BDP**

The compounds used in the present study consist of a group of six **TAPY–bodipy (TAPY-BDP)** conjugates, depicted in Fig. 2, along with two model compounds for comparison. One of the model compounds is a bodipy derivative bearing the TPP vector, while the other is a molecule lacking any targeting element (bearing only a propyl group). The TPP derivative serves to evaluate the mitochondrial targeting efficacy of the TAPY conjugates, while the propyl-substituted compound helps assess whether the bodipy core alone exhibits any inherent mitochondrial localization. Among the six TAPY conjugates, the structural differences lie in the nature of the R substituent. All compounds were synthesized and characterized according to the literature [55].

The aim of the following studies is to quantitatively assess the fluorescence intensity produced by each molecule under varying conditions in cultures of two fungal species: *C. albicans* and *P. kudriavzevii* (formerly *C. krusei*).

Firstly, *C. albicans* was used as a model organism to evaluate **TAPY-BDP** derivatives as potential mitochondrial stains, together with the corresponding model compounds **TPP-BDP** and **propyl-BDP**. Cultures of *C. albicans* were incubated with the fluorescent probes and subsequently imaged by confocal laser scanning microscopy (CLSM) at different concentrations. In a preliminary screening, concentrations of 1 µM or higher produced saturated images that were unsuitable for resolving subcellular details, whereas 500 nM afforded images with well-defined intracellular structures.

**Fig. 3:**
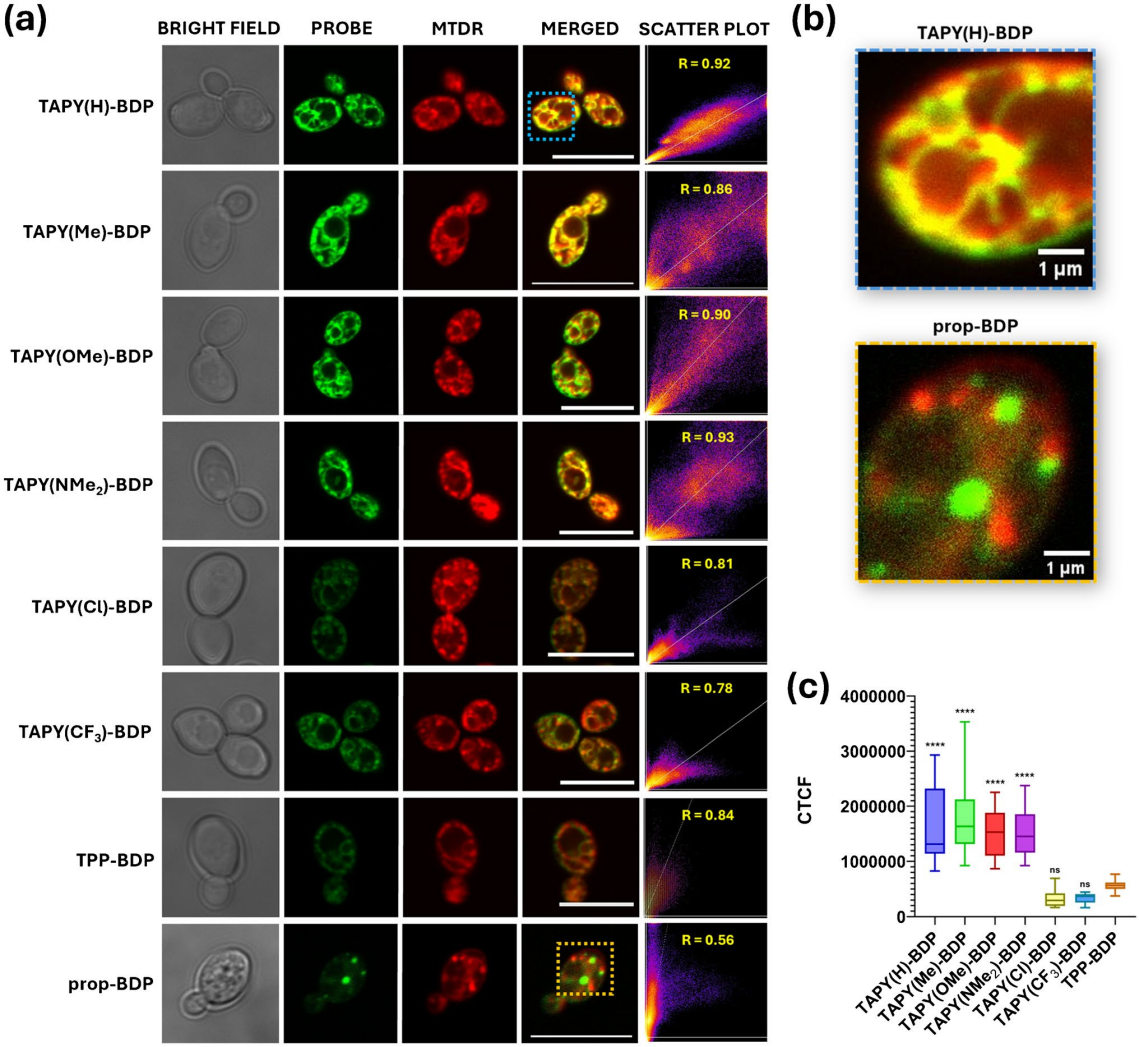
**(a)** Confocal laser scanning microscopy (CLSM) images of *C. albicans* cells (CECT 1394) incubated with 500 nM of the six **TAPY–BDP** conjugates, **TPP–BDP**, and **prop–BDP**, with 100 nM of MTDR for 30 min at 37 °C. Channels are displayed in the following order: bright field, probe channel (λ_ex_ 488 nm), MTDR channel (λ_ex_ 633 nm), merged images, and pixel-by-pixel scatter plots showing Pearson correlation coefficients (*R*) with MTDR. Scale bar: 10 µm. **(b)** Magnified CLSM images highlighting the regions indicated in panel (a), illustrating the distribution pattern of **TAPY(H)–BDP** and **prop–BDP**. Scale bar: 1 µm. **(c)** Corrected Total Cell Fluorescence (CTCF) quantification for cells treated with the six **TAPY–BDP** derivatives and **TPP–BDP**, measured in the green channel (λ_ex_ 488 nm). Data are presented as median ± interquartile range (IQR), *n* = 20. Statistical analysis was performed using one-way ANOVA followed by Dunnett’s multiple comparisons test against **TPP–BDP**. Significance levels: ****p ≤ 0.0001, ***p ≤ 0.001, **p ≤ 0.01, *p ≤ 0.05, ns = not significant

To determine the subcellular localization of the probes, the cultures were co-incubated with the well-established mitochondrial dye MitoTracker™ Deep Red (MTDR). Comparison of the fluorescence collected in the green channel (bodipy-containing compounds) and the red channel (MTDR) indicated that the spatial distribution of MTDR and all BDP-based probes, with a single exception, was very similar. As shown in Fig. 3a, the green and red channels, together with their merged images, yield yellow–orange signals consistent with extensive colocalization. The exception is **propyl-BDP**, which displays a clearly distinct distribution pattern. These observations indicate that both TAPY and TPP vectors efficiently direct the bodipyfluorophore to mitochondria, as expected, although notable differences in signal intensity were observed (see below). Colocalization was quantified using the Pearson correlation coefficient (PCC), which was high for the TAPY and TPP probes (0.78–0.93), but significantly lower for the propyl derivative (PCC = 0.56). Visually, the difference between a mitochondria-targeted fluorophore such as **TAPY(H)-BDP** and **propyl-BDP** is evident in Fig. 3b, where the latter shows a characteristic scattering of non-overlapping green and red puncta. The precise subcellular localization of **propyl-BDP** lies beyond the scope of this work, although it may correspond to lipidic bodies within the cell, in agreement with the distribution observed in our recent publication [55].

One of the aims of this study was to quantify the cellular uptake of TAPY derivatives in comparison with the TPP “gold standard” and, in addition, to determine whether a particular substituent R on the TAPY scaffold confers an advantage for mitochondrial vectorization. Quantitative analysis of the CLSM images allowed us to calculate the corrected total cell fluorescence (CTCF), i.e., the total fluorescence from samples containing *n* = 20 cells. This analysis (Fig. 3c) shows that TAPY probes bearing R = H, Me, OMe, or NMe_2_ produce higher fluorescence intensities than those with R = Cl or CF_3_. More importantly, these four TAPY derivatives stain the mitochondria of *C. albicans* much more efficiently than **TPP-BDP**. In our previous report, we showed that one of the TAPY systems afforded a stronger signal than the corresponding TPP model compound in a kinetic assay using MCF7 cells, which is consistent with the present findings. At this stage, however, these results should not be overgeneralized. The physicochemical factors that govern cellular internalization and subsequent mitochondrial targeting, most notably charge and hydrophobicity, are numerous and require careful balance [60–63]. Equally important, each cell line has its own specific characteristics that may cause the same probe to behave differently. In other words, biological factors are as critical as physicochemical ones and must be considered on an equal footing.

**Fig. 4:**
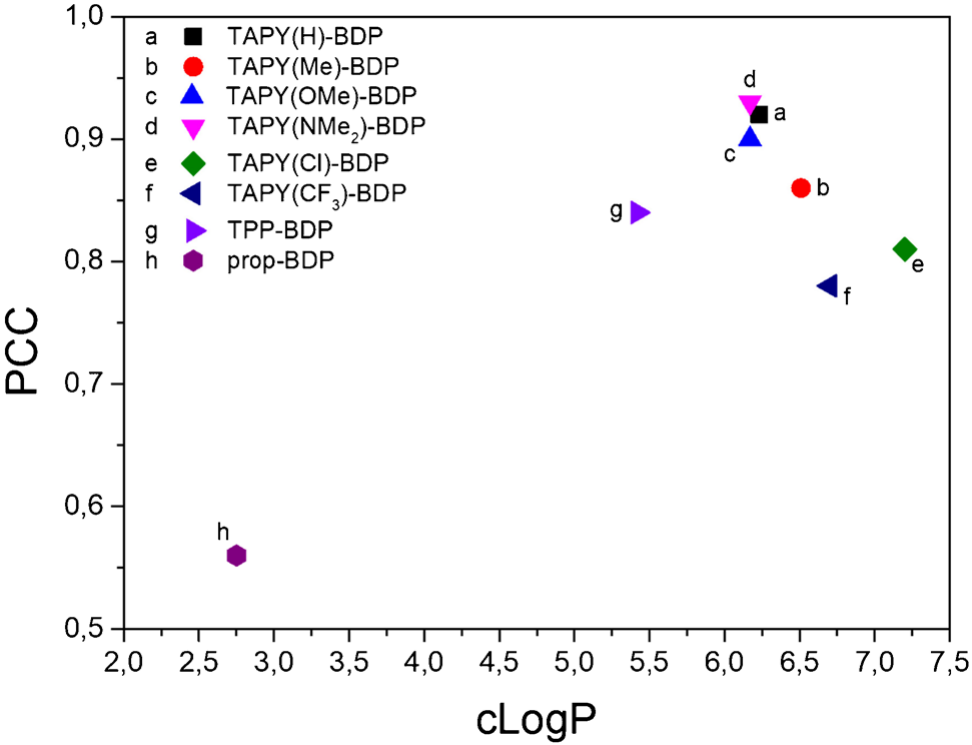
Relationship between calculated clogP and Pearson correlation coefficients (PCC) for mitochondrial colocalization

The lower performance of TAPY derivatives bearing R = Cl and CF_3_ can be tentatively attributed to two factors. On the one hand, and probably more important, the electron-withdrawing nature of these substituents, which disfavors delocalization of the positive charge within the TAPY scaffold. On the other hand, the higher lipophilicity of Cl and CF_3_ may render the conjugates excessively hydrophobic for optimal mitochondrial targeting. To further explore this possibility, we examined how PCC vary as a function of clogP within the TAPY series (Fig. 4). The more lipophilic Cl- and CF_3_-substituted dyads appear as a distinct group with consistently lower signals. Although this correlation does not establish a mechanistic cause/effect relationship, it reveals a trend within the series, suggesting that lipophilicity may modulate the mitochondrial targeting efficiency of TAPY conjugates in yeast cells.

**Fig. 5:**
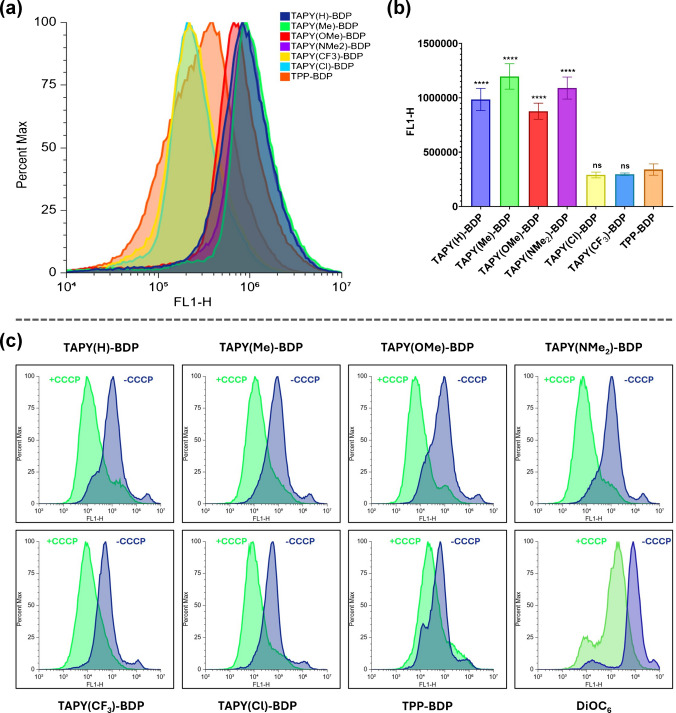
**(a)** Flow cytometry histograms (FL1-H) of cultures of *C. albicans* cells (CECT 1394) treated with six **TAPY–BDP** derivatives and **TPP–BDP** (500 nM) showing green fluorescence intensity detected in FL1 (λ_ex_ 488 nm; λ_em_ 533/30 nm). Data are displayed as normalized frequency (Percent Max). (**b)** Quantification of FL1-H fluorescence from three independent biological replicates (mean ± SD). One-way ANOVA followed by Dunnett’s test was used to compare each **TAPY–BDP** derivative with **TPP–BDP** (****p < 0.0001; ns = not significant). **(c)** Independent assay to test the dependency of the uptake upon the polarization of the mitochondrial membrane: fluorescence intensities of probes in the presence (+ CCCP) or absence (–CCCP) of 50 µM CCCP, showing fluorescence reduction consistent with loss of mitochondrial membrane potential. All probes were compared under identical conditions, and DiOC_6_ was included as a positive (potential-dependent) control

The CLSM-based analysis described above was complemented by flow cytometry (FC) using the same *C. albicans* cultures and probes at identical concentrations. All fungal samples efficiently internalized the TAPY dyads, although the fluorescence intensity varied markedly among the different molecular probes. Figure 5a presents fluorescence histograms for all TAPY derivatives and the TPP model compound, highlighting their differential uptake. A direct numerical comparison of the mean fluorescence intensities for all mitochondrial dyes is provided in Fig. 5b. In agreement with the CLSM results, FC data indicate that R = H, Me, OMe, and NMe_2_ perform better compared to R = Cl and CF_3_ for designing TAPY-based mitochondrial probesand, notably, outperform the TPP reference compound.

Accumulation within mitochondria is generally driven by the ability of cationic dyes to concentrate across the highly polarized inner mitochondrial membrane (high negative membrane potential), as previously indicated. In order to test whether this is the case with **TAPY-BDP** dyes, in an independent assay, it was used the membrane depolarizer carbonyl cyanide 3-chlorophenylhydrazone (CCCP). As can be seen in Fig. 5c, the signals obtained in the presence of CCCP were significantly lower than those without this additive. In the same assay, a positive control like DiOC_6_ was also tested [59].

### Studies with *Candida albicans* at 100 nM (probe concentration)

Once it was established that some TAPY derivatives are superior mitochondrial dyes compared to others and, in particular, outperform the TPP derivative, the next question was whether these probes could also stain yeast mitochondria at very low concentrations. To address this, a colocalization assay analogous to the previous one was carried out using **TAPY(OMe)-BDP**, **TPP-BDP**, and **propyl-BDP**, but at a fivefold lower probe concentration.

**Fig. 6:**
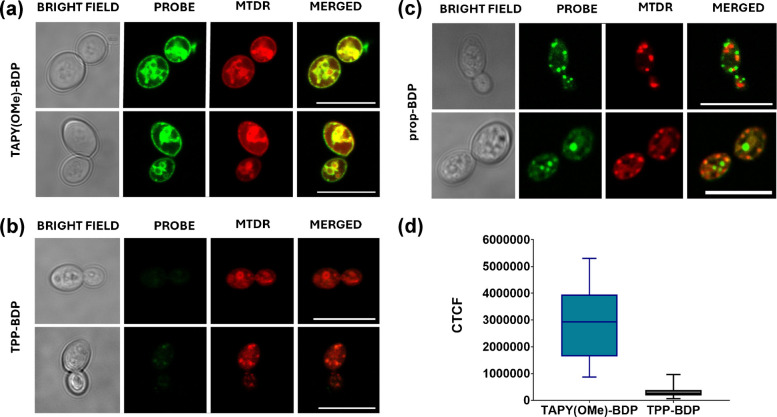
Confocal laser scanning microscopy (CLSM) images of *C. albicans* cells (CECT 1394) incubated with 100 nM **TAPY(OMe)–BDP** (**a**), **TPP–BDP** (**b**), and **prop–BDP** (**c**), together with 100 nM MitoTracker Deep Red^FM^ (MTDR), for 30 min at 37 °C. Images are shown as bright field, probe channel (λ_ex_ 488 nm), MTDR channel (λ_ex_ 633 nm), and merged images. Scale bar: 10 µm. **(d)** Corrected Total Cell Fluorescence (CTCF) quantification comparing **TAPY(OMe)–BDP** and **TPP–BDP** under identical conditions

The images in Fig. 6 show that the **TAPY(OMe)-BDP** derivative still provides a clear fluorescence signal and closely matches the MTDR staining pattern, confirming its mitochondrial localization, whereas the propyl derivative does not. The most striking observation, however, is the very low fluorescence intensity of the TPP-containing molecule under these conditions, almost undetectable. This unexpected result further underscores the usefulness of TAPY-based probes for imaging *C. albicans* mitochondria at low concentrations. A detailed mechanistic explanation for the low TPP signal lies beyond the scope of this work, but one plausible hypothesis is the action of efflux pumps that expel TPP derivatives more efficiently than TAPY conjugates. Indeed, TPP-based compounds have frequently been reported in the literature as excellent substrates for this class of membrane proteins, both in mammalian [64] and fungal cells [65].

### Studies with *Pichia kudriavzevii* at 100 nM (probe concentration)

**Fig. 7:**
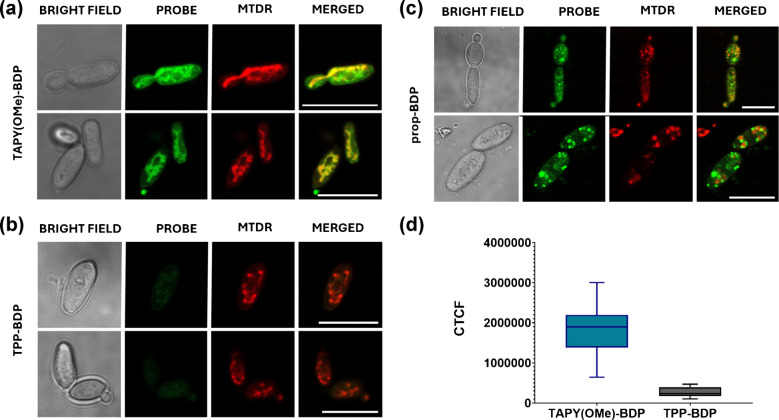
Confocal laser scanning microscopy (CLSM) images of *P. kudriavzevii* (CECT 19105) incubated with 100 nM **TAPY(OMe)–BDP** (**a**), **TPP–BDP** (**b**), and **prop–BDP** (**c**), together with 100 nM MitoTracker Deep Red FM (MTDR), for 30 min at 37 °C. Images are shown as bright field, probe channel (λ_ex_ 488 nm), MTDR channel (λ_ex_ 633 nm), and merged images. Scale bar: 10 µm. **(d)** Corrected Total Cell Fluorescence (CTCF) quantification comparing **TAPY(OMe)–BDP** and **TPP–BDP** under identical conditions

Next, we examined whether other fungal species were also amenable to mitochondrial staining with TAPY conjugates at such low concentrations, and compared their performance with the TPP and propyl model compounds. For this purpose, *P. kudriavzevii* (traditionally referred to as *C. krusei*) was selected. This microorganism has only recently been reassigned to a different genus and displays notable metabolic differences from *C. albicans*. Thus, the comparison is particularly relevant, especially considering their distinct responses to azole antifungals (*P. kudriavzevii* being intrinsically resistant) [66]. The colocalization assay described above was therefore repeated using this yeast, and the results are shown in Fig. 7. Once again, **TAPY(OMe)-BDP** exhibited higher staining efficiency than the TPP derivative and a distribution pattern clearly distinct from that of **propyl-BDP**. These results further support the main conclusion of this work, namely that TAPY constitutes a promising vector for targeting fungal mitochondria.

## Conclussion

A quantitative comparison of six **TAPY-BDP** fluorescent dyads as mitochondrial markers in fungi was carried out using CLSM and FC. With *C. albicans* as a model organism, the fluorescence intensity obtained at 500 nM for TAPY derivatives bearing H, Me, OMe, or NMe_2_ substituents was higher than that observed for the Cl- and CF_3_-substituted analogues when evaluated by CLSM. The same trend was confirmed by FC.

Crucially, these TAPY conjugates outperformed the TPP counterpart as mitochondrial markers, positioning the TAPY scaffold as a potential competitive alternative to TPP in certain contexts, although more studies are needed to establish the advantages and drawbacks of the new carriers. Experiments conducted at lower probe concentrations (100 nM) further reinforced this conclusion and even accentuated the contrast, at least in the case of **TAPY(OMe)-BDP** versus **TPP-BDP**, in both *C. albicans* and *P. kudriavzevii*.

The data presented here introduces new fluorescent probes for bioanalytical studies in pathogenic fungi, which are urgently needed given the emerging threat posed by azole-resistant strains. In addition, we are confident that higher-quality experimental data obtained from living fungi will help improve the performance of current deep-learning–based predictive approaches [67].

Furthermore, considering that mitochondrial targeting vectors can also deliver therapeutics into cells, as recently demonstrated for cationic TPP and benzamidine derivatives [68–73], TAPY-based carriers may likewise be exploited in the future for targeted delivery, potentially with comparable or even superior performances.

## Supplementary Information

Below is the link to the electronic supplementary material.

Supplementary file1 The Supporting Information includes the full statistical analysis of fluorescence measurements, with normality and variance tests, post hoc comparisons, and residual diagnostics. (PDF 512 KB)

## Data Availability

Data are available from the corresponding author upon reasonable request.
