# Supplementary material for: Fluorescent TAPY–bodipy dyads as tools for imaging fungal mitochondria by confocal microscopy and flow cytometry
Source: Anal Bioanal Chem. 2026 Feb 26;418(9):2745–56. doi: 10.1007/s00216-026-06404-6 (PMC13079517; doi:10.1007/s00216-026-06404-6)
Supplement: Supplementary file 1 — Supplementary file1 The Supporting Information includes the full statistical analysis of fluorescence measurements, with normality and variance tests, post hoc comparisons, and residual diagnostics. (PDF 512 KB) [file 216_2026_6404_MOESM1_ESM.pdf]

Supporting Information

Fluorescent TAPY–bodipy dyads as tools for imaging fungal mitochondria by confocal microscopy and flow cytometry

Jean C. Neto,[a] Rosa de Llanos,[b] Francisco Galindo*[a]

[a] *Universitat Jaume I de Castellón, Departamento de Química Inorgánica y Orgánica, Avda. Vicente Sos Baynat s/n, 12071 Castellón de la Plana, Spain.*

[b] *Unidad Predepartamental de Medicina, Universitat Jaume I de Castellón, Avda. Vicente Sos Baynat s/n, 12071 Castellón de la Plana, Spain.*

* E-mail: francisco.galindo@uji.es

Table of Contents

Table S1. Summary of one-way ANOVA assumptions and test results....2

Table S2. One-way ANOVA followed by Dunnett's multiple comparisons test....3

Figure S1. Residual analysis of fluorescence quantification using CTCF values....3

Table S3. Summary of Kruskal–Wallis test results.....4

Table S4. Dunn's post hoc test following Kruskal–Wallis analysis....4

Table S1. Summary of one-way ANOVA assumptions and test results. Includes Brown-Forsythe and Bartlett's tests for homogeneity of variances, ANOVA table, and residual normality tests (Anderson-Darling, D'Agostino-Pearson, Shapiro-Wilk, Kolmogorov-Smirnov). This table reports the dataset presented in Figure 3c (Main Manuscript).

one-way ANOVA	ANOVA COMPARISON
Brown-Forsythe test	
F (DFn, DFd)	9,265 (6, 133)
P value	<0,0001
P value summary	****
Are SDs significantly different (P < 0.05)?	Yes

Bartlett's test	
Bartlett's statistic (corrected)	117,2
P value	<0,0001
P value summary	****
Are SDs significantly different (P < 0.05)?	Yes

ANOVA table	SS	DF	MS	F (DFn, DFd)	P value
Treatment (between columns)	5,12343E+13	6	8,53904E+12	F (6, 133) = 47,81	P<0,0001
Residual (within columns)	2,37524E+13	133	1,78589E+11		
Total	7,49867E+13	139			

Normality of Residuals

Test name	Statistics	P value	Passed normality test (alpha=0,05)?	P value summary
Anderson-Darling (A2*)	2,511	<0,0001	No	****
D'Agostino-Pearson omnibus (K2)	29,8	<0,0001	No	****
Shapiro-Wilk (W)	0,9367	<0,0001	No	****
Kolmogorov-Smirnov (distance)	0,1349	<0,0001	No	****

Data summary	
Number of treatments (columns)	7
Number of values (total)	140

Table S2. One-way ANOVA followed by Dunnett's multiple comparisons test**.** Comparison of each TAPY-BDP compound against TPP-BDP (control). Mean fluorescence values (n = 20), confidence intervals, and adjusted p-values are shown. This table reports the dataset presented in Figure 3c (Main Manuscript).

one-way ANOVA	MULTIPLE COMPARISON
Number of families	1
Number of comparisons per family	6
Alpha	0,05

Dunnett's multiple comparisons test	Mean Diff,	95,00% CI of diff,	Significant?	Summary	Adjusted P Value	G-?	
TPP-BDP vs. TAPY(H)-BDP	-1049206	-1396706 to -701707	Yes	****	<0,0001	A	TAPY(H)-BDP
TPP-BDP vs. TAPY(Me)-BDP	-1215401	-1562901 to -867902	Yes	****	<0,0001	B	TAPY(Me)-BDP
TPP-BDP vs. TAPY(OMe)-BDP	-937298	-1284797 to -589798	Yes	****	<0,0001	C	TAPY(OMe)-BDP
TPP-BDP vs. TAPY(NMe_2)-BDP	-960970	-1308470 to -613471	Yes	****	<0,0001	D	TAPY(NMe_2)-BDP
TPP-BDP vs. TAPY(Cl)-BDP	250087	-97412 to 597587	No	ns	0,2525	E	TAPY(Cl)-BDP
TPP-BDP vs. TAPY(CF_3)-BDP	232513	-114987 to 580013	No	ns	0,3199	F	TAPY(CF_3)-BDP

Test details	Mean 1	Mean 2	Mean Diff,	SE of diff,	n1	n2	q	DF
TPP-BDP vs. TAPY(H)-BDP	566021	1615227	-1049206	133637	20	20	7,851	133
TPP-BDP vs. TAPY(Me)-BDP	566021	1781423	-1215401	133637	20	20	9,095	133
TPP-BDP vs. TAPY(OMe)-BDP	566021	1503319	-937298	133637	20	20	7,014	133
TPP-BDP vs. TAPY(NMe_2)-BDP	566021	1526992	-960970	133637	20	20	7,191	133
TPP-BDP vs. TAPY(Cl)-BDP	566021	315934	250087	133637	20	20	1,871	133
TPP-BDP vs. TAPY(CF_3)-BDP	566021	333508	232513	133637	20	20	1,74	133

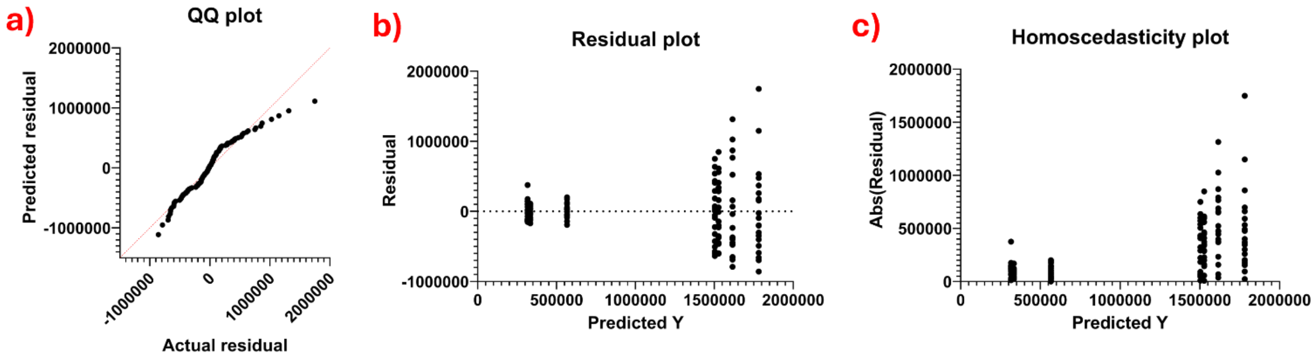

Figure S1. Residual analysis of fluorescence quantification using CTCF values. a) QQ plot of the residuals. b) Residual vs. predicted values plot (residual plot). c) Homoscedasticity test, Bartlett's test for homogeneity of variances. This table reports the dataset presented in Figure 3c (Main Manuscript).

Table S3. Summary of Kruskal–Wallis test results. Includes p-value, test statistic, number of groups, and total number of values analyzed. This table reports the dataset presented in Figure 3c (Main Manuscript).

Kruskal-wallis test	ANOVA results
Table Analyzed	Data 3 - AJUSTADO
Kruskal-Wallis test	
P value	<0,0001
Exact or approximate P value?	Approximate
P value summary	****
Do the medians vary signif. (P < 0.05)?	Yes
Number of groups	7
Kruskal-Wallis statistic	108,4
Data summary	
Number of treatments (columns)	7
Number of values (total)	140

Table S4. Dunn's post hoc test following Kruskal–Wallis analysis**.** Displays mean rank differences, significance levels, adjusted p-values, and comparison details between TPP-BDP and TAPY derivatives. This table reports the dataset presented in Figure 3c (Main Manuscript).

Kruskal-wallis test	Multiple comparisons results
Number of families	1
Number of comparisons per family	6
Alpha	0,05

Dunn's multiple comparisons test	Mean rank diff,	Significant?	Summary	Adjusted P Value	G-?	
TPP-BDP vs. TAPY(H)-BDP	-51,05	Yes	***	0,0004	A	TAPY(H)-BDP
TPP-BDP vs. TAPY(Me)-BDP	-58,85	Yes	****	<0,0001	B	TAPY(Me)-BDP
TPP-BDP vs. TAPY(OMe)-BDP	-49,45	Yes	***	0,0007	C	TAPY(OMe)-BDP
TPP-BDP vs. TAPY(NMe_2)-BDP	-50,65	Yes	***	0,0005	D	TAPY(NMe_2)-BDP
TPP-BDP vs. TAPY(Cl)-BDP	27,05	No	ns	0,2096	E	TAPY(Cl)-BDP
TPP-BDP vs. TAPY(CF_3)-BDP	25,45	No	ns	0,2833	F	TAPY(CF_3)-BDP

Test details	Mean rank 1	Mean rank 2	Mean rank diff,	n1	n2
TPP-BDP vs. TAPY(H)-BDP	48	99,05	-51,05	20	20
TPP-BDP vs. TAPY(Me)-BDP	48	106,9	-58,85	20	20
TPP-BDP vs. TAPY(OMe)-BDP	48	97,45	-49,45	20	20
TPP-BDP vs. TAPY(NMe_2)-BDP	48	98,65	-50,65	20	20
TPP-BDP vs. TAPY(Cl)-BDP	48	20,95	27,05	20	20
TPP-BDP vs. TAPY(CF_3)-BDP	48	22,55	25,45	20	20
